# Commencement Week at the Baltimore College of Dental Surgery

**Published:** 1869-04

**Authors:** 


### ARTICLE V.

### *Commencement Week at the Baltimore College of Dental Surgery.*

The examinations of the candidates for the degree of "Doctor of Dental Surgery" were concluded at noon Saturday, Feb. 27th, and the result was announced to the class assembled in the Chemical Hall, at 5 P.M. the same day.

On Monday, March 1st, the series of lectures, known as the "Harris Lectures," commenced with some general remarks on "Dental Physiology," by Prof. W. H. Atkinson of New York.

At 2 P.M. the second lecture of the series was delivered by Dr. John Allen of New York, the subject of his remarks being "Mechanical Dentistry." To the regret of all, Dr. Allen, immediately after the delivery of his lecture, was compelled to leave for Cincinnati, owing to the receipt of a telegram informing him of the death of a near relative. We have the promise of Dr. Allen's interesting lecture for publication in the *Journal.*

Dr. B. F. Arrington of Wilmington N.C. was also present and would have followed Dr. Allen, had not news arrived which compelled him to leave at once for home. The class, therefore, was deprived of the pleasure of hearing an address which, we feel assured by our knowledge of Dr. Arrington's ability as a lecturer, would have proved highly interesting and instructive.

Dr. Atkinson, in the absence of Dr. Arrington, occupied the 3 o'clock hour with the third lecture of the series, a very interesting one, on "Mallet Filling;" also the 4 and 5 o'clock hours.

From 8 to 11 A. M. and from 2 to 3 P. M. on Tuesday, Dr. Atkinson held Clinics in the Infirmary, at which he demonstrated very thoroughly the use of the "mallet."

At 11 A. M. Tuesday, Dr. Lemorcier of Paris delivered the seventh lecture of the series, the subject of his remarks being "Clastic Models." Dr. L. used to illustrate his lecture some of the most beautiful and perfect models of the brain and other portions of the nervous system, teeth of different animals, &c., it has ever been our good fortune to behold; and the familiarity of the lecturer with his subject and preparations, displayed a remarkable knowledge of anatomy and physiology.

An account of this lecture, by Professor Noel, will appear in a future number of this *Journal.*

At 3 P. M. Tuesday, immediately after Dr. Atkinson's second Clinic, Dr. S. H. Williams, formerly of Alexandria,

Va. but now a resident of Baltimore, delivered the eighth lecture of the series, his subject being "Filling Teeth," which was attentively listened to and appreciated by the class.

Dr. Atkinson followed Dr. Williams with two more lectures on the "Legal Relations of the Profession," which contained much good advice to the members of the graduating class.

Drs. W. H. Morgan of Nashville, Tenn. and F. Y. Clark of Savannah, Geo., were confidently expected, and much regret was expressed at their inability to be present and add to the instruction and pleasure afforded by these lectures.

*Commencement Exercises.* On the evening of the 3d of March, the *Twenty Ninth Annual Commencement* was held in the Concordia Opera House. The hall and galleries of this immense building were crowded with a large and appreciative concourse of ladies and gentlemen, many of the former bringing with them boquets of choice flowers for their friends among the graduates.

The Blues' Band under the direction of Prof. Holland, was present, and interspersed the exercises with fine music.

After the faculty, graduates and guests had entered and taken their seats on the stage, the exercises were commenced with prayer by the Rev. W. E. Muncey, D.D. of the Methodist Episcopal Church South. The dean of the College, Prof. F. J. S. Gorgas, then read the authority of the College for conferring the degrees, announced the names of the graduates, and also the changes in the Faculty since the last annual commencement. The degree of Doctor of Dental Surgery was then conferred by the dean upon the following gentlemen, twenty-six in number:

Charles Louis George Becht, M.D. ........... Holland.
Alonzo Goold Bouton ........................... Georgia.
David Clark Card, M.D. ........................ Connecticut.
James A. Chapman .............................. Virginia.
Abner Franklin Claywell ....................... Tennessee.
Thomas C. Edwards ............................. Tennessee.

Reuben Kennerly George.............................Virginia.
John P. H. Grant.................. ............Tennessee.
Charles Emmet Hammen............................Virginia.
A. Frank Herr.............................. ......Pennsylvania.
James B. Hodgkin.....................................Virginia.
Jacob Zollinger Hoffer..............................Pennsylvania.
George Fisk Keesee..................................Virginia.
Benjamin Franklin Kidd..............................Virginia.
George Hamilton Kirk, Jr.............................Alabama.
William Eppa Norris.................................Maryland.
Joseph Nevin Rentch.................................West Virginia.
John Walter Scribner.................................Virginia.
Emanuel F. Shuler.....................................Mississippi.
John C. Storey, M.D............ .....................Virginia.
Singleton Townshend................,..........Maryland.
Luther Trump............................................Maryland.
William Wallace Westmoreland...............Alabama.
Richard Baily Winder...............................Virginia.
George H. Winkler.... ...........................South Carolina.
Judson Boardman Wood.........................Virginia.

Of which number there were from

Maryland 3; Virginia 10; West Virginia 1; South Carolina 1; Tennessee 3; Alabama 2; Georgia 1; Mississippi 1; Pennsylvania 2; Connecticut 1; Holland 1.

After the degrees were conferred, the Valedictory Address was delivered by Prof. E. Lloyd Howard, M.D. We present it to our readers in the present number of the *Journal*; it contains sound and practical advice and was highly appreciated by the class.

It is proper to remark that the Valedictory Address was to have been delivered by the late Prof. Piggot.

Dr. G. F. Keesee of Richmond Va., a member of the graduating class, then replied with a well written and properly delivered address, which was received by all present with great satisfaction. This address also appears in the present number of the *Journal*.

Professor Thomas E. Bond then arose and apologizing for departing from the order of the exercises, read to the audience a letter from the wife of the late Professor Piggot, in which she said that on the evening following his departure for Virginia she found lying on his desk the fragments of a valedictory address intended to be delivered this evening, and which she remarked, although only broken fragments, were like a mirror broken to pieces, each particular fragment still reflecting with renewed brilliancy.

Professor Bond then read these fragments which were exceedingly beautiful and interesting, after which he paid a feeling tribute to their late colleague, with whom he said he had had the extreme pleasure of enjoying an intimate acquaintance for many years, and regarded him as one of the most talented and polished type of the true gentleman and scholar it had ever been his fortune to meet with.

After these remarks by Professor Bond the audience was dismissed with the benediction by the Rev. Dr. Muncey.

